# Formation Mechanism and Dispersion of Pseudo-Tetragonal BaTiO_3_-PVP Nanoparticles from Different Titanium Precursors: TiCl_4_ and TiO_2_

**DOI:** 10.3390/ma11010051

**Published:** 2017-12-29

**Authors:** Jinhui Li, Koji Inukai, Yosuke Takahashi, Akihiro Tsuruta, Woosuck Shin

**Affiliations:** 1 Department of Frontier Materials, Nagoya Institute of Technology, Nagoya 466-8555, Japan; kinki-ri@aist.go.jp; 2 R & D Center, Noritake Co., Ltd., Miyoshi 470-0293, Japan; koujiinukai@n.noritake.co.jp (K.I.); yosuke-takahashi@n.noritake.co.jp (Y.T.); 3 Inorganic Functional Material Research Institute AIST, Nagoya 463-8560, Japan; a.tsuruta@aist.go.jp

**Keywords:** BaTiO_3_, poly(vinylpyrrolidone), hydrothermal, dispersion, c/a

## Abstract

Nano-sized tetragonal BaTiO_3_ (BT) particles that are well dispersed in solution are essential for the dielectric layer in multilayer ceramic capacitor technology. A hydrothermal process using TiCl_4_ and BaCl_2_, as source of Ti and Ba, respectively, or the precursor TiO_2_ as seed for the formation of BT, and poly(vinylpyrrolidone) (PVP) as a surfactant, was employed in this study to enhance both the dispersibility and tetragonality (c/a) simultaneously in a single reaction process. The process parameters, i.e., the ratio of TiO_2_ substitution of TiCl_4_, the reaction time, and PVP content were systematically studied, and the growth mechanism and relation between the tetragonality and the particle size are discussed. Dynamic light scattering (DLS) analysis was used to show that truncated pseudo-tetragonal BT-PVP particles with an average size of 100 nm, having a narrow size distribution and a coefficient of variation (CV) as low as 20% and being mono-dispersed in water, were produced. The narrow particle size distribution is attributed to the ability of PVP to inhibit the growth of BT particles, and the high c/a of BT-PVP to heterogeneous particle growth using TiO_2_ seeds.

## 1. Introduction

Miniaturization and capacity enlargement of multilayer ceramic capacitors (MLCCs) are essential for the next generation of electronic devices; thus, the dielectric material used in these MLCCs requires thinner dielectric layers and a grain size below a few hundred nanometers [1]. BaTiO_3_ (BT) is the most important dielectric oxide material [2] and its industrial synthesis occurs by way of a solid-phase reaction involving high-temperature sintering. However, because this manufacturing procedure is not suitable for small particle sizes below a few hundred nanometers, many solution processes for the fabrication of BT have been attempted [3]. In addition, owing to their good biocompatibility and unique properties, BT nanoparticles have potential biomedical applications [4,5]. 

A hydrothermal process is one of the most popular approaches to fabricate metal oxide nanoparticles directly from solution at elevated temperatures ranging from 100 to 280 °C and high pressures to improve the crystallinity of the products [6]. Hydrothermally synthesized BT powder has a superfine particle size and narrow particle size distribution with good crystallinity of the pseudo-tetragonal phase. However, the solution process leads to low tetragonality, resulting in low dielectric constants, for which there are two reasons: (1) the size effect [7] reflecting a thermodynamic phase transition due to isotropic pressure and (2) the nonstoichiometric defects of BT [8]. This size effect of BT is considered to be unavoidable for small particles, irrespective of the powder synthesis route that is employed. In reactions in aqueous solution, the hydroxyl lattice defect is induced, and is often detrimental to the dielectric properties and causes the device quality to deteriorate.

BT prepared by a hydrothermal method forms a tetragonal core and cubic surface layer and this is related to the particle size, with the critical size of transformation of cubic-BaTiO_3_ to tetragonal-BaTiO_3_ reported to be 80 nm or larger, for example by Xu et al. [9]. In this method, a number of parameters, such as the solvent, raw materials for BaTiO_3_, temperature, soaking time, concentration, and pressure can be adjusted. Several attempts were made to investigate the effect of the reaction solvent to obtain fine tetragonal BT. Alcohol-based solvents such as ethanol, methanol, and *n*-propanol yielded nano-sized cubic-phase powders. Kwon et al. [10] reported partially tetragonal BT powder using ethanol as a solvent. Similar studies were conducted by Habib et al. [11].

Furthermore, a drawback of nano-sized BT particles is aggregation and poor dispersibility in solution, especially in the case of highly concentrated suspensions. To date, many methods including the use of a surfactant, pH control, and ultrasonication have been attempted and among them, surface modification is the most common approach to increase the dispersibility of nanoparticles in the solvent [12,13]. Recently, Hai et al. [14] prepared highly dispersed BT-PVP at a low temperature with TiCl_4_, BaCl_2_, KOH, and poly(vinylpyrrolidone) (PVP) in aqueous solution. The method, which succeeded in solving the problem of a fine particle size and dispersion in one step, was motivated by the core-shell particle process proposed by Izu et al. [15]. Other authors attempting to obtain smaller high-dispersion nanoparticles prepared highly dispersed 80-nm-sized cubic BT-PVP particles by controlling the reaction parameters and the metal ion concentration in solution [16,17].

Two well-known approaches were proposed in the form of an in-situ reaction and dissolution-precipitation reaction for the growth of BT in a hydrothermal process [16]. To understand which one prevails and which one leads to high-quality BT, the use of various sources of Ti and the ratio of Ti to Ba were studied, with TiO_2_ frequently used as the source of Ti raw material [18,19].

In this study, the fabrication of BT-PVP with controlled dispersibility was attempted with the aim of producing a truncated or cubic-shaped pseudo-tetragonal structure and to realize the uniform dispersion of nanoparticles in aqueous solution. Among the aforementioned process parameters, we focused on the substitution of the Ti source as raw material to prepare pseudo-tetragonal BT-PVP nanoparticles as the most important parameter and proposed to use TiO_2_ nanoparticles as seed material. The size and shape dependences of BT nanoparticles on the reaction time and the PVP content were then investigated.

## 2. Results and Discussion

### 2.1. Enhanced Tetragonality by TiO_2_ Substitution

**Figure 1:**
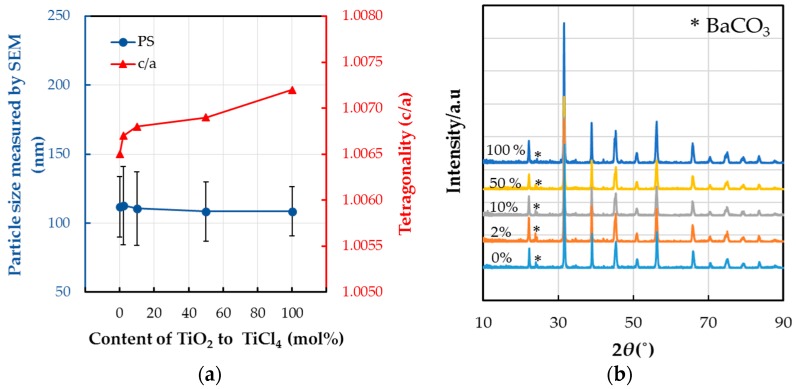
(**a**) The effect of TiO_2_ substitution on the size of particles measured by field emission scanning electron microscopy (FE-SEM) and the c/a of pseudo-tetragonal crystallites estimated from the (**b**) XRD pattern corresponding to the two reflection peaks from the (200) and (002) planes. The standard deviation of the particle size, which is shown as vertical bars, decreases as the TiO_2_ substitution increases.

This section presents our investigation of the effects of different Ti sources, namely TiCl_4_ and TiO_2_, on the growth and crystallinity of the produced BT-PVP nanoparticles. As introduced before, although the effect of an organic solvent such as EtOH can improve the tetragonality of the BT nanoparticles, in this study, in order to assess the effect of the Ti source, an aqueous solution was prepared without organic solvent. The synthesis parameters of the source material are 0.32 M BaCl_2_·2H_2_O, either TiCl_4_ or TiO_2_ corresponding to a 0.2 M Ti source, 100 g/L PVP, and 2.3 M KOH, under the reaction conditions of 230 °C for 24 h, which were optimized in previous research [20]. Figure 1a shows the change in the mean particle size (PS) of BT-PVP that was prepared by substituting the raw material TiCl_4_ by TiO_2_, and the tetragonality, the c/a ratio of the pseudo-tetragonal structure. The X-ray diffraction (XRD) results indicate that the phase of the BT-PVP nanoparticles is pseudo-tetragonal and that it is independent from the Ti source. The tetragonality of BT is confirmed by the diffraction peak in the 2*θ* region of 44–46°, as shown in Figure 1b. 

The particle size of BT-PVP decreased slightly with the substitution of TiO_2_ in the solution, but did not deviate much from the size of 110 nm. However, the increase in tetragonality, c/a, to values over 1.007 as a result of increased TiO_2_ substitution is very noticeable, even though the particle size decreased. In principal, the effect of the size of BT nanoparticles is that a smaller particle size leads to lower tetragonality, but in this result, the trend changed for the different sources of Ti. This difference is first reported here, and the presence of PVP polymer in the solution might be responsible for achieving a higher dielectric constant of BT with TiO_2_ as the source. This result is very promising for producing high-quality dielectric films or coatings fabricated using these BT particles with PVP surfaces.

The different reaction mechanisms caused the size of the particles to be different, and the BT-PVP obtained by using TiO_2_ shows a slightly smaller particle. The process presented herein for the synthesis of BT-PVP nanoparticles using TiO_2_ is similar to the respective two-step methods proposed by Wada et al. [21] and Joung et al. [22]. In the latter case, cubic BT particles were synthesized at 100 °C and subsequently heated to high temperatures of 260 °C for 60 h to fabricate BT nanoparticles with the size of approximately 95 nm and a high c/a ratio of 1.0081, but some TiO_2_ remained and was not entirely consumed [23]. Han et al. [23] also reported that high c/a is 1.0083 as the particle size of BT is 126 nm, but reduced to 1.0081 as the particle size is 149 nm, which is not explained clearly. In this study, as shown in the XRD pattern in Figure 1b, no TiO_2_, which exhibits peaks at 27.4°, remained in all the samples. The presence of BaCO_3_, with peaks at 27.7°, was observed for the BT-PVP with high TiO_2_ content. Detailed data of the XRD profile, which the *y*-axis shows in log-scale, are plotted in Figure S2. The c/a of BT-PVP is 1.0072, and the particle size is 108 nm, which was prepared with TiO_2_ for 24 h. The formation of BaCO_3_ is ascribed to the interaction of Ba ions with CO_2_ species, which occurs both in atmospheric air and in the reaction solution (this byproduct could be easily removed by washing the obtained product with dilute hydrochloric acid). Considering this impurity, the amount of Ba source material was considered to be sufficient, contrary to other research that was carried out with a higher Ba to Ti ratio [22,23].

Nucleation can occur either homogeneously from bulk solution or heterogeneously at the surface of crystalline TiO_2_ or related amorphous phases. Since homogeneous nucleation requires relatively high supersaturation conditions, it is more likely to occur when highly reactive and relatively soluble hydrous Ti gel is involved. In this study, however, our understanding was that the heterogeneous process prevails, and relatively small particles of high tetragonality are grown with the help of TiO_2_ substitution. The featuring property of BT-PVP synthesized in this study compared to others is the dispersion of BT nanoparticles in the solution, as discussed again later. Furthermore, it is a noticeable fact that the size distribution of the BT-PVP particles is very narrow, as shown in Figure 1a by the deviation bars, and the coefficient of variation (CV) of the BT-PVP approximates 20.0%. As discussed in our previous reports [14], the PVP absorbed on the BT surface is considered to inhibit further particle growth, and the deviation in particle size is reduced by increasing the oxide substitution.

### 2.2. Dependency of the Formation Mechanism and Morphological Changes on the Reaction Time

**Figure 2:**
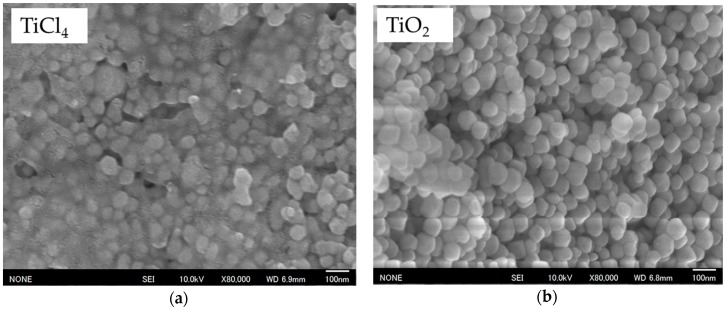
FE-SEM images of hydrothermally synthesized BT-PVP particles using different Ti sources for 5 h: (**a**) by TiCl_4_; (**b**) by TiO_2_.

The reaction time of BT-PVP particles was shortened to 5 h (as opposed to 24 h) and Figure 2 shows FE-SEM (JSM–6335FM, JEOL Ltd., Tokyo, Japan) images comparing the morphology of BT-PVP synthesized by different sources of Ti. The particle size of BT-PVP prepared using TiCl_4_ as the source is smaller and the morphology is irregular compared to that prepared using TiO_2_ as source. The tetragonality (c/a) of BT-PVP is 1.0012 and 1.0045 when prepared by TiCl_4_ and TiO_2_, respectively. This demonstrates that increased TiO_2_ substitution enhances the formation of tetragonal crystals. In addition, it can be seen that the particles are covered with gel in Figure 2a, but not in Figure 2b, and the amount of dispersant adsorbed on the BT surface is 4.09 and 2.28 wt % respectively, measured by thermogravimetric analysis (TG), indicating that the 5 h reaction time is still too short to decompose PVP completely when TiCl_4_ is used as the source. In our previous study [20], BT-PVP prepared by a hydrothermal process demonstrated high dispersion because PVP completely decomposed as the reaction time became longer, and the dispersion worsened as the reaction time shortened because the PVP was incompletely decomposed as measured by Fourier transform infrared spectroscopy (FT-IR, FT/IR-610, JASCO Corp., Tokyo, Japan). The dispersibility of the particles prepared by both sources was less than satisfactory, but as the reaction time was extended to 24 h, good dispersion was obtained. 

**Figure 3:**
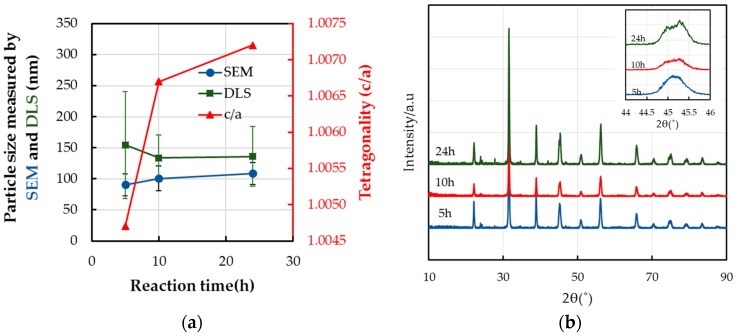
Variation in the (**a**) size and c/a and (**b**) XRD peaks of BT-PVP particles as a function of the reaction time (5, 10, and 24 h) at 230 °C. The inset is the XRD magnification from 44 to 45°.

Figure 3 shows the effect of the reaction time on the particle size and the tetragonality, the c/a, of BT-PVP prepared by adding TiO_2_ to the starting solution. The size of BT-PVP particles estimated by observation using FE-SEM is not increased much with reaction time. The tetragonality of BT is confirmed by the diffraction peak in the 2*θ* region of 44−46°, as shown in Figure 3b. The XRD pattern shows the formation of BaCO_3_ and the absence of TiO_2_. However, an evaluation of the dispersion property of the BT-PVP by dynamic light scattering (DLS) measurement, as shown in Figure 3a, indicates that the average particle size analyzed by DLS is reduced from 150 to 135 nm as the reaction time increases, which means the dispersion improves, i.e., the particle size approaches that determined by FE-SEM. Considering that the size of the BT-PVP particles measured by FE-SEM ranges from 90 to 130 nm, the synthesized BT-PVP particles are regarded as being mono-dispersed after the 24 h reaction.

**Figure 4:**
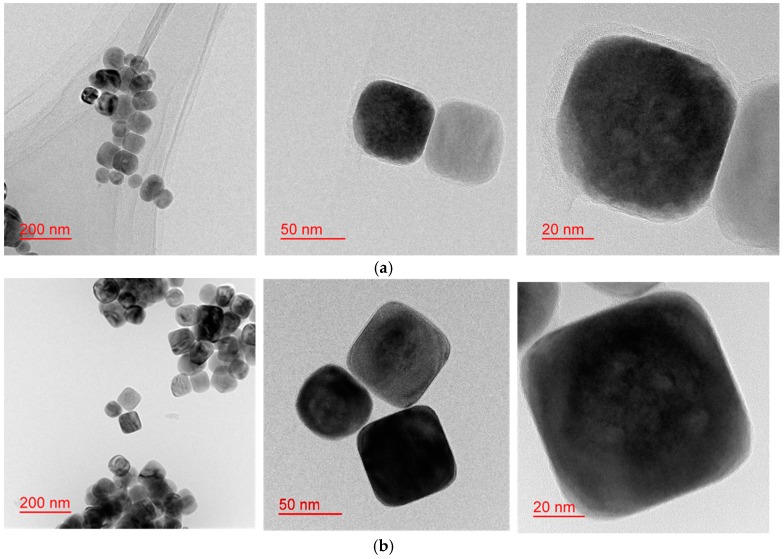

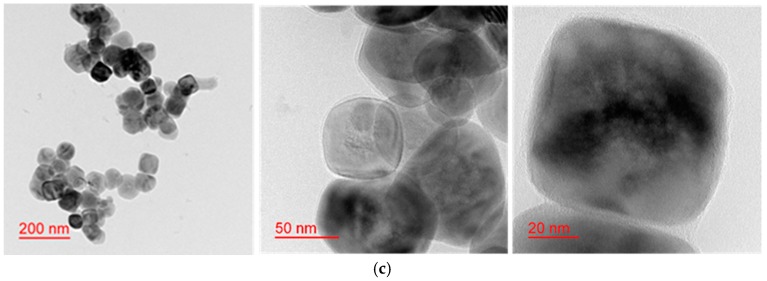
BT-PVP particles prepared at 230 °C for 24 h from different Ti sources, (**a**) TiCl_4_; and (**b**) TiO_2_, respectively; (**c**) BT-PVP particles prepared by TiO_2_ at 230 °C for 5 h.

As the reaction time reached 24 h, the shape of the synthesized BT-PVP particles clearly became cubic (Figure 4a,b). Furthermore, the surface of the BT-PVP particles is coated by PVP, which is a prominent difference from the BT-PVP synthesized at atmospheric pressure. Although the size of the particle is not affected much by changing the reaction time, the shape of the nanocrystals is different. The particles prepared by TiO_2_ are truncated cubic to a larger extent, but those prepared by TiCl_4_ are more rounded with more defects. The morphology of these particles, compared with those shown in Figure 4c, becomes more cubic, and the particle size slightly increases as reaction time increases. 

For the highly dispersed BT-PVP, the thickness of the PVP layer adsorbed on the BT surface was approximately 3 nm and is reduced compared to that of BT-PVP after a 5 h reaction as shown in Figure 4c. The change in the particle size in the reaction of core-oxide shell-PVP type was discussed previously by Izu [24] and Li [17] who varied the concentration and molecular weight of PVP and determined that a rich PVP solution induces smaller dispersed particles. However, in our work, an intermediate PVP concentration of 100 g/L was selected to prohibit unwanted reactions such as the gelation of PVP. The effect of the PVP content is discussed below.

### 2.3. Dispersion of BT-PVP and the Effect of PVP Concentration

**Figure 5:**
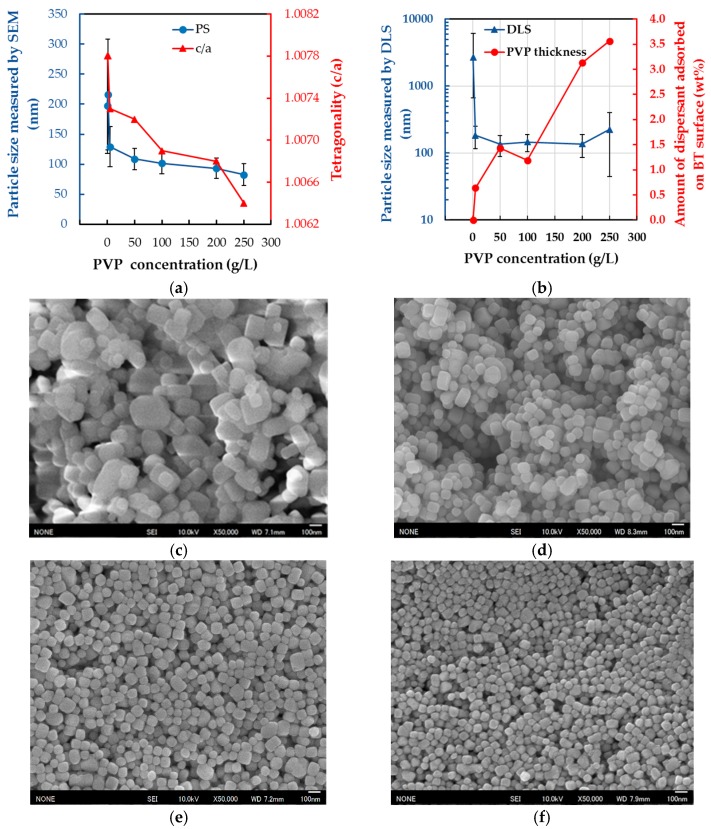
Effect of PVP concentration on (**a**) the PS and the c/a (**b**) the dispersion and PVP shell thickness. The standard deviation of the particle size is shown as vertical bars in (a,b). FE-SEM image of BT-PVP prepared with different PVP concentrations: (**c**) no PVP; (**d**) 5 g/L; (**e**) 100 g/L; (**f**) 250 g/L.

Figure 5 shows the effect of the PVP concentration on the size of BT particles, PS, the tetragonality, c/a, and the dispersion, i.e., the particle size evaluated by DLS. The particle size decreases from 210 to 70 nm when the PVP concentration increases from 0 to 250 g/L, and the tetragonality (c/a) also decreases, as shown in Figure 5a. This indicates that the presence of PVP in increasing concentrations prohibits particle growth and, as a result, affects the particle tetragonality. Izu et al. [24] reported the same result, i.e., that PVP prohibits the particle growth because the viscosity of the reaction solution increases with increasing PVP concentration. Considering the different lengths of the error bars indicating the standard deviation of the particle size, the size distribution of BT-PVP without PVP is broader, but becomes narrow owing to the addition of PVP. 

We previously reported that the PVP decomposes with increasing reaction temperature and time in the hydrothermal process [20]. The highly dispersed state of BT-PVP is maintained by the decomposed PVP absorbed on the BT surface via electrostatic repulsion. The high degree of dispersion is maintained constant at approximately 5–250 g/L of the PVP concentration as shown in the DLS results in Figure 5b. The amount of dispersant adsorbed on the BT surface, as estimated by TG analysis, increases with increasing PVP concentration. Concurrently, the particle size as measured by SEM decreases from 130 to 80 nm. It is speculated that the total surface area increases with decreasing particle size, thereby resulting in an increase in the amount of PVP dispersant on the BT surface.

Based on this result, the amount of the decomposed PVP absorbed on the surface of BT is considered to vary with the PVP concentration, such that a high degree of dispersion is maintained. However, a higher PVP content of 200 g/L induced aggregation, which is understood to be due to the unwanted polymerization of the PVP as discussed before [17]. Detailed data of the DLS profile are plotted in Figure S1. Figure 5c–e show images of the BT-PVP particles prepared at 0, 5, 100, and 200 g/L PVP concentrations, respectively. Without the PVP, the particles have irregular shapes; however, the particle size distribution of BT-PVP prepared with PVP becomes narrow, and the shape becomes cubic. As discussed in our previous report, the PVP can prohibit the diffusion of cations, and not only reduce the size of the BT particles, but also ensure that the deviation in size remains small. This size distribution control with the PVP is successfully demonstrated again in this study using TiO_2_ as the source.

**Figure 6:**
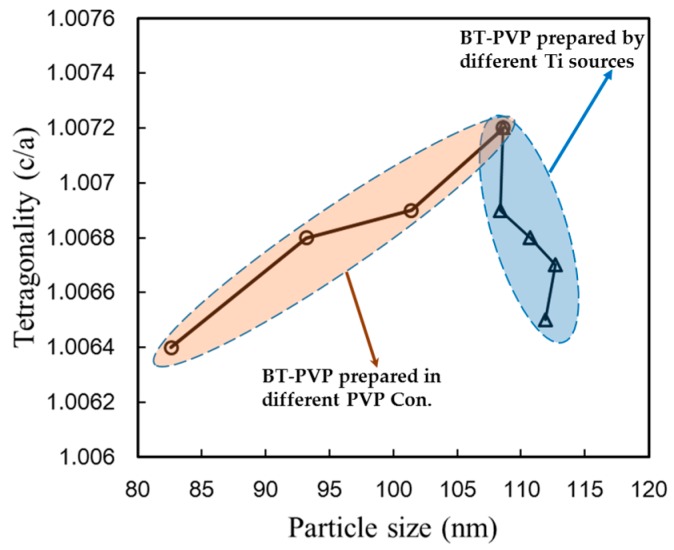
Relation of the tetragonality, c/a, and the particle size of BT-PVP nanoparticles with the process variables of the PVP concentration and Ti source. The blue- and orange-colored regions contain the plotted data of BT-PVP particle sizes obtained from Figure 1 and Figure 5, respectively.

Figure 6 shows the tetragonality as a function of the particle size. The tetragonality values of all the data are in very good agreement with the declining trend according to the decrease in the particle size reported by Lee et al. [25]. This trend is caused by the presence of a cubic shell layer, which is considerable in nano-size BT and is also accounted for in the model by Hoshina et al. [26]. It is notable that one novel advance in this study is that the BT-PVP particles are mono-dispersed as a result of the PVP on the cubic BT surface, which suppressed growth of the BT particles to narrow the size distribution of the particles. The orange-colored area in Figure 6 shows the effects of the PVP content, and this strongly supports the mechanism of decreased BT particle size and tetragonality by PVP. The second important achievement in this study is that the effect of the Ti source was to induce different structural defects during the growth of the BT nanoparticles in the PVP-containing hydrothermal process; as a result, the c/a of BT-PVP in this study increased by oxide substitution. 

## 3. Materials and Methods

**Figure 7:**
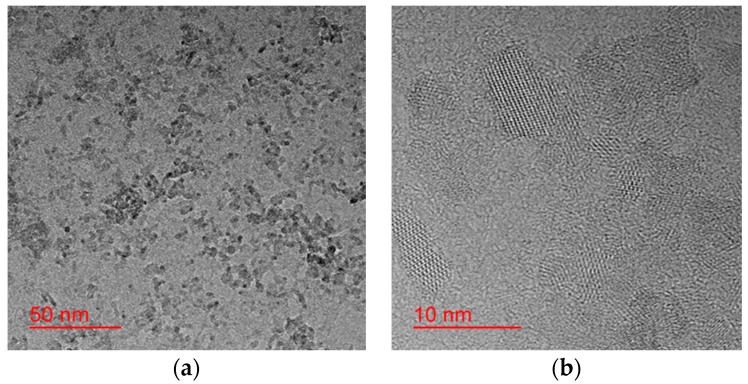
Microscope images of the TiO_2_ seed crystals as the substituted Ti source: (**a**) the low-magnification image shows the narrow particle size distribution; (**b**) the high-resolution image shows that the particles are single crystals.

Titanium oxide particle powder (TiO_2_) was used as the seed crystal material. Barium chloride dihydrate (BaCl_2_·2H_2_O, 90%), potassium hydroxide (KOH, 85%) mineral agent (Wako Pure Chemical Industries Ltd., Tokyo, Japan), and PVP dispersant (Sigma-Aldrich, St. Louis, MO, USA) of a molecular weight of 10,000 g/mol were used as precursors. TiO_2_ particles of the order of several nm are used as an alternative source of Ti^4+^ in the place of TiCl_4_ and their shapes are shown in Figure 7. The lower magnification image shows a narrow particle size distribution, and the high-resolution image shows that the particles are single crystals. 

The inset shows an image of the reaction vessel including a 50 mL Teflon container and an autoclave. First, an aqueous solution containing 0.32 M of BaCl_2_·2H_2_O and the TiO_2_ particles corresponding to 0.2 M of the Ti source, 100 g/L of PVP, and 2.3 M of KOH were mixed in the 50 mL Teflon container. The reaction solution was prepared such that the total volume equaled approximately 40 vol % of the 50 mL Teflon container.

The solution mixture was sealed and placed into the autoclave and heated at the specified reaction temperature and time. After the reaction, the container was cooled to room temperature and the resulting white precipitate was extracted by using a centrifugal separator (Himac CR20GII, Hitachi, Tokyo, Japan). The precipitate was washed with distilled water and ethanol, and then dried at 60 °C for 24 h to obtain the BT-PVP particles. The detailed reaction conditions utilized in this study are summarized in Table 1.

The crystalline phase of the obtained BT-PVP particles was examined by X-ray diffraction (XRD, Smartlab Rigaku Corp., Tokyo, Japan, Cu Kα 1.5405 Å, 30 mA/40 KV, scan speed 20°/min, with 2*θ* ranging from 10–90°). The tetragonality of the BT-PVP particles was estimated by measuring the peak of the (200) plane again by reducing the scan speed to 0.01°/min for the range 44–46°, and the scan result was fitted by the Gaussian function. The XRD pattern was deconvoluted into the two peaks of the (200) and (002) planes, respectively, for the lattice parameters of c and a, as reported by Kwon et al. [10]. The tetragonality of the produced BT-PVP nanoparticles was quantified by the value of c/a. The particle morphology was observed via field emission scanning electron microscopy (FE–SEM, JSM–6335FM, JEOL Ltd., Tokyo, Japan) and transmission electron microscopy (TEM, JEM 1000 K, JEOL Ltd., Tokyo, Japan).

The sizes and size distributions of the BT-PVP particles were determined via image analysis performed by using the specialized SmileView software. Dynamic light scattering analysis (DLS, FPAR–10001, Otsuka Electronics Co., Ltd., Osaka, Japan) was used to evaluate the dispersibility of 1 wt % particles in aqueous solutions by conducting five sequential measurements. The amount of dispersant adsorbed on the surface the BT particles was estimated by measuring the weight loss from 200 to 600 °C, as determined by thermogravimetric analysis (TG; TG–DTA2010SA, Bruker AXS K.K., Tokyo, Japan).

## 4. Conclusions

This paper reports our study of the hydrothermal process in which BaTiO_3_ (BT) particles were produced using the source compounds TiO_2_ as the seed oxide and PVP as the surfactant. Both high dispersibility in water and high tetragonality (or c/a) of the nanoparticles were achieved in a simple process facilitated by the PVP and TiO_2_ seed particles. The formation mechanism of this hydrothermal BT-PVP was investigated by systematically varying the process parameters including the ratio of TiO_2_ substitution of TiCl_4_, PVP content, and reaction time, to determine their effect on the BT-PVP properties. The results showed that truncated pseudo-tetragonal BT-PVP particles with an average particle size of 100 nm with a narrow size distribution, CV of 20%, and high dispersibility in water were obtained, as confirmed by DLS analysis. 

The narrow size distribution of the BT-PVP particles was explained in terms of the suppression of the growth of the BT particles by the PVP, and the high c/a of the BT-PVP particles is attributed to heterogeneous growth with the TiO_2_ seeds. Both the high dispersion of nanoparticles in aqueous solution and the high c/a of BT-PVP are highly promising properties for various industrial applications.

## Figures and Tables

**Table 1:** Reaction conditions utilized during the BT-PVP synthesis in this study.

Reaction Parameter	TiO_2_ (mol %)	Reaction Time (h)	PVP Concentration (g/L)
Content of TiO_2_ in TiCl_4_	0, 1, 2, 10, 50, 100	24	50
Reaction time	100 (TiO_2_)	5, 10, 24	50
	0 (TiCl_4_)	5, 24	
PVP concentration	100	24	0, 1, 5, 50, 100, 200, 250

## Supplementary Materials

The following are available online at www.mdpi.com/1996-1944/11/1/51/s1. Figure S1: Effect of PVP concentration in the reaction solution on the dispersion of BT-PVP particles in water, as evaluated by DLS. Figure S2: XRD diffraction of BT-PVPs prepared with different content of TiO_2_.
