# Supplementary material for: Formation Mechanism and Dispersion of Pseudo-Tetragonal BaTiO3-PVP Nanoparticles from Different Titanium Precursors: TiCl4 and TiO2
Source: Materials (Basel). 2017 Dec 29;11(1):51. doi: 10.3390/ma11010051 (PMC5793549; doi:10.3390/ma11010051)
Supplement: Supplementary file 1 [file materials-11-00051-s001.docx]

Supporting Information

Formation mechanism and dispersion of pseudo-tetragonal BaTiO_3_-PVP nanoparticles from different titanium precursors: TiCl_4_ and TiO_2_

Jinhui Li ^1, 3^, Koji Inukai ^2^, Yosuke Takahashi ^2^, Akihiro Tsuruta ^3^ and Woosuck Shin ^1, 3^ *

^1^ Department of Frontier Materials, Nagoya Institute of Technology, Nagoya 466-8555, Japan; kinki-ri@aist.go.jp (J. L)

^2^ R&D Center, Noritake Co., Ltd., Miyoshi 470-02, Japan; yosuke-takahashi@n.noritake.co.jp (Y. T.); koujiinukai@n.noritake.co.jp (K. I.)

^3^ Inorganic Functional Material Research Institute AIST, Nagoya 463-8560, Japan; w.shin@aist.go.jp (W.S.); a.tsuruta@aist.go.jp (A.T.)

***** Correspondence: [w.shin@aist.go.jp](mailto:w.shin@aist.go.jp) (W.S.); Tel.: +81-52-736-7107

| 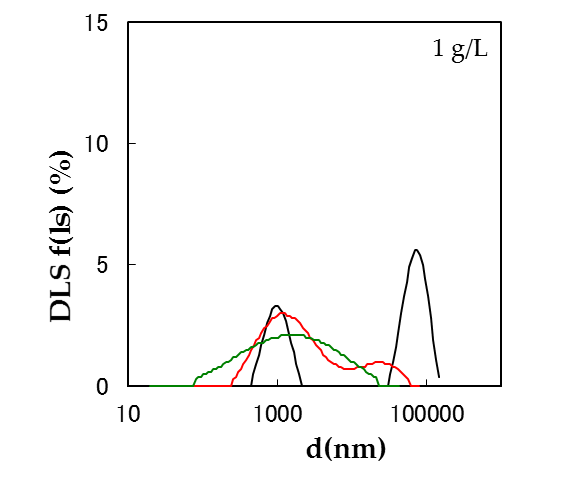 | 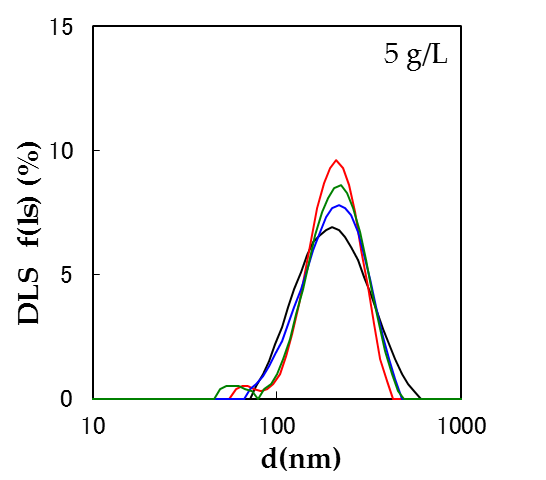 |
| --- | --- |
| 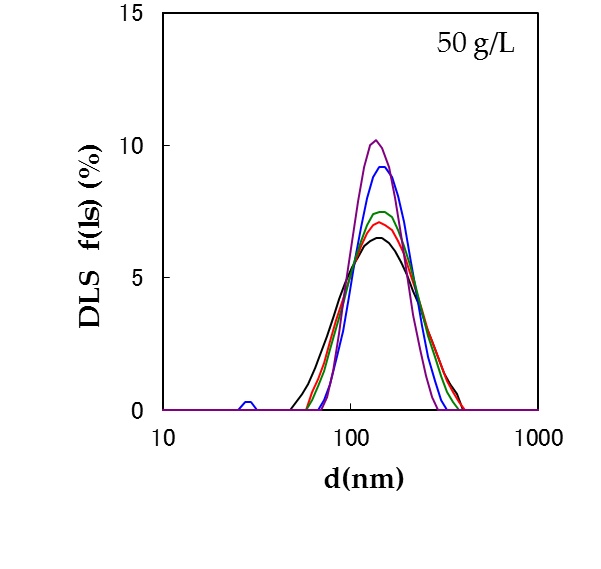 | 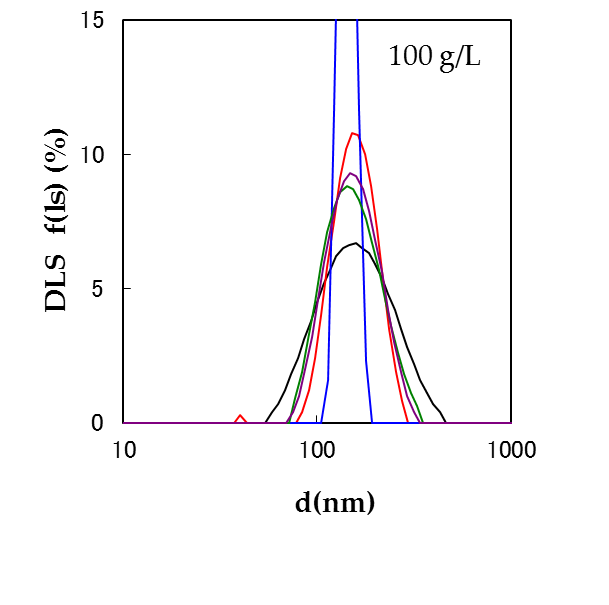 |
| 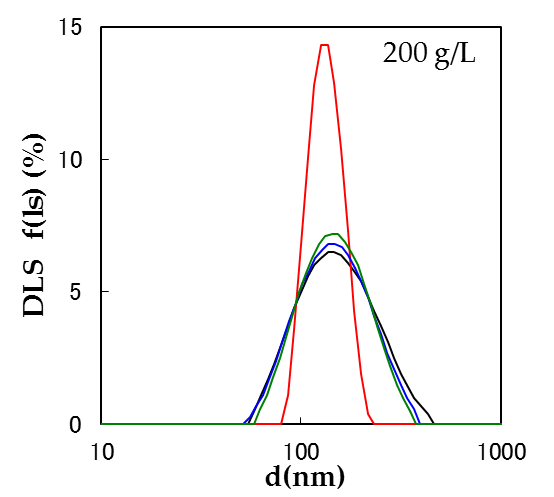 | 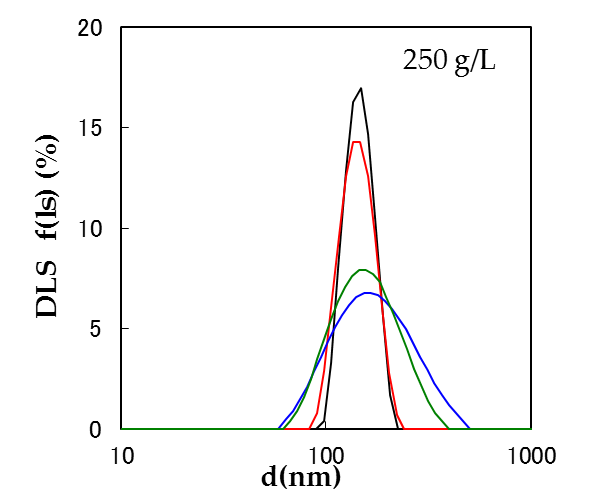 |

Figure S1: Effect of PVP concentration in the reaction solution on the dispersion of BT-PVP particles in water, as evaluated by DLS.

| 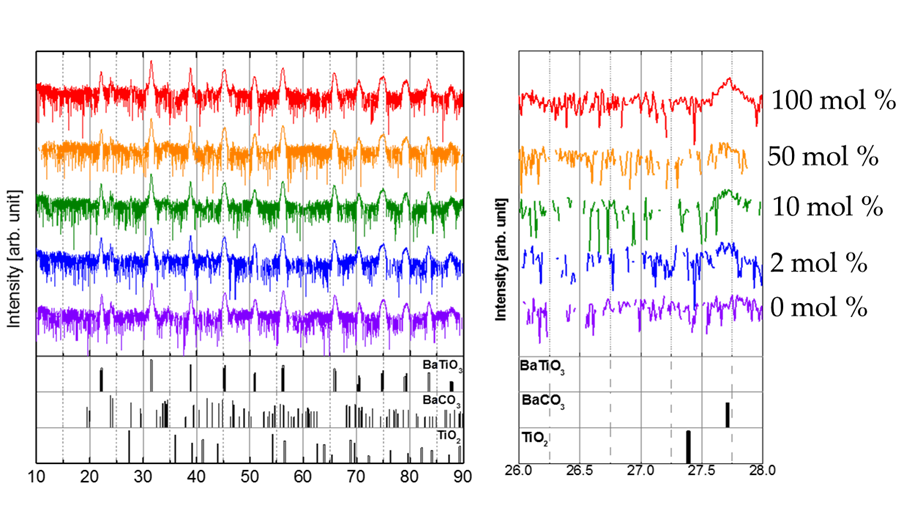 |
| --- |

Figure.S2: XRD diffraction of BT-PVPs prepared with different content of TiO_2_.

© 2017 by the authors. Submitted for possible open access publication under the
terms and conditions of the Creative Commons Attribution (CC-BY) license (http://creativecommons.org/licenses/by/4.0/).
